# Association Between Abdominal Adipose Tissue Distribution and Obstructive Sleep Apnea in Chinese Obese Patients

**DOI:** 10.3389/fendo.2022.847324

**Published:** 2022-03-24

**Authors:** Bingwei Ma, Yingying Li, Xingchun Wang, Lei Du, Shilin Wang, Huihui Ma, Donglei Zhou, Taofeek Usman, Liesheng Lu, Shen Qu

**Affiliations:** ^1^ Department of Endocrinology and Metabolism, Shanghai Tenth People’s Hospital, School of Medicine, Tongji University, Shanghai, China; ^2^ Department of Gastrointestinal Surgery, Shanghai Tenth People’s Hospital, Tongji University, Shanghai, China; ^3^ School of Pharmacy, East China University of Science and Technology, Shanghai, China; ^4^ Thyroid Research Center of Shanghai Tenth People's Hospital, Shanghai, China; ^5^ Division of Endocrinology and Diabetes, Department of Pediatrics, Children’s Hospital of Pittsburgh of University of Pittsburgh Medical Center (UPMC), University of Pittsburgh School of Medicine, Pittsburgh, PA, United States; ^6^ Department of Hepatobiliary Surgery, Shanghai Tenth People’s Hospital, Tongji University, Shanghai, China

**Keywords:** OSAS, obesity, visceral adipose deposit, BIA, fat distribution

## Abstract

**Purpose:**

Factors related to the occurrence of obstructive sleep apnea syndrome (OSAS) in obesity have not been fully clarified. The aim of this study was to identify the association between OSAS and abdominal fat distribution in a cohort of Chinese obese patients.

**Methods:**

This cross-sectional study collected demographic data of 122 obese patients who were admitted into the in-patient unit of the Department of Endocrinology, Shanghai Tenth People’s Hospital from July 2018 to January 2021. OSAS was diagnosed based on the results of overnight polysomnography, and the abdominal fat distribution was measured by bioelectrical impedance analysis (BIA). Univariate and multivariate logistic regression analyses were used to investigate the association between OSAS and the distribution of abdominal fat.

**Results:**

(1) The mean age (SD) of the obese patients included was 32.44 (11.81) years old, and the overall incidence rate of OSAS was 51.06%. Twenty-four (25.53%) patients had mild OSAS, 10 (10.64%) had moderate OSAS, and 14 (14.89%) had severe OSAS. The apnea hypopnea index (AHI) of men was significantly higher than that of women (5.50, interquartile range (IQR) 3.80–30.6 vs. 4.2, IQR 1.4–12 events/h, *p* = 0.014). Meanwhile, men had a significantly higher visceral fat area when compared with women (180.29 ± 51.64 vs. 143.88 ± 53.42 cm^2^, *p* = 0.002). (2) Patients with OSAS had a significantly higher waist circumference, fasting plasma glucose, 2 h postprandial plasma glucose, glycated hemoglobin, and visceral fat area than patients without OSAS (all *p* < 0.05). (3) AHI was significantly positively associated with BMI, neck circumference, waist circumference, and visceral fat area (*r* = 0.306, *p* = 0.003; *r* = 0.380, *p* < 0.001; *r* = 0.328, *p* = 0.002; *r* = 0.420, *p* < 0.001) but not with subcutaneous fat area (*p* = 0.094). Multivariate analysis demonstrated that abdominal fat area and fasting plasma glucose were independent risk factors for OSAS (odds ratio, 1.016; 95% confidence interval, 1.005–1,026, *p* = 0.005; odds ratio, 1.618; 95% confidence interval, 1.149–2.278, *p* = 0.006).

**Conclusions:**

In obese patients, the abdominal visceral adipose deposit but not the subcutaneous fat area was associated with OSAS and was an independent risk factor for OSAS. Therefore, improving the distribution of abdominal fat may contribute to alleviating the severity of OSAS in obesity.

## Introduction

Due to the development of the society and changes in the lifestyle, the prevalence of obesity has increased significantly among the global population (1). Obesity as a disease has become a global public health problem and is associated with several other diseases, such as type 2 diabetes mellitus (T2DM), hypertension, nonalcoholic fatty liver disease, and obstructive sleep apnea syndrome (OSAS) (2). With regard to OSAS, obesity is one of the most important risk factors, and its prevalence is increasing in parallel with the severity of obesity (3, 4). In addition, OSAS also promotes weight gain (5). Consequently, obesity and OSAS interact with each other. However, the risk factors related to OSAS in obesity has not been fully clarified.

OSAS is characterized by repetitive upper airway obstruction during sleep, recurrent oxygen desaturation, and frequent arousal from sleep (6). The overall population prevalence ranges from 9% to 38% and is higher in men. It increases with age, and in some elderly groups, the incidence of OSAS was reported to be as high as 90% in men and 78% in women (7). OSAS not only decreases the quality of life (QOL) of the patients but also increases the societal burden (8). Several studies have shown that OSAS is associated with numerous adverse health outcomes such as hypertension, cognitive impairment, stroke, and Alzheimer’s disease (9, 10). A new meta-analysis has demonstrated that blood pressure control can benefit from the treatment of OSAS (11). OSAS is typically prevalent in middle-aged or older adults (12); however, younger patients with obesity are also prone to OSAS (13).

The mechanism underlying the occurrence of OSAS is not fully understood. Multifactorial issues including craniofacial changes, alteration in upper airway muscle function, pharyngeal neuropathy, and fluid shift towards the neck, as well as obesity may cause OSAS (12). OSAS results from a combination of anatomic features that narrow the upper airway along with the permissive effect of insufficient neuromuscular compensation during sleep. With an increasing volume of the upper airway structures, the severity of OSAS also increases (14). In addition, abdominal adipose deposits due to obesity may be a causative factor for OSAS because of decreased lung volume and traction on the pharynx (15).

Research on abdominal adipose deposits and OSAS is relatively limited. No study has explored the relationship of abdominal fat distribution and OSAS. We hypothesized that visceral adiposity and/or subcutaneous fat play an important role in the improvement of OSAS. Therefore, we designed this study to further illuminate their relationship.

## Materials and Methods

This cross-sectional study enrolled 94 obese patients from the in-patient unit of the Department of Endocrinology, Shanghai Tenth People’s Hospital between July 2018 and January 2021. Obesity was diagnosed as body mass index (BMI) over 30 kg/m^2^. Inclusion criteria were as follows (1): obesity with BMI ≥30 kg/m^2^ and (2) aged 18~65 years old. The exclusion criteria were as follows (1): underlying diseases such as severe liver injury, decompensated liver cirrhosis, kidney failure, cardiac ischaemic disease, and malignancies (2); history with upper airway surgery (3); gestation (4); passive smoking or alcoholism within the last three years (5); active treatment for other respiratory disorders; and (6) taking sleeping pills. The study was approved by the ethics committee of Shanghai Tenth People’s Hospital. All participants included in the study provided written informed consent. All patients enrolled underwent an examination for the abdominal fat distribution and overnight polysomnography.

### Data Collection

In this study, we collected demographic data including age, sex, height, weight, and BMI. We also recorded the circumference of the neck, waist, and hip, which were measured by professional staff as previously published (16). Systolic blood pressure and diastolic blood pressure data were collected and recorded. Before measuring the blood pressure, all patients were asked to rest for 10 min and to avoid smoking and drinking coffee or tea. Every anthropometric measurement was performed twice, and the average was chosen as the final recording to reduce error. Additionally, venous blood samples were collected after overnight fasting for over 8 h. The measurement of glucose metabolism including fasting plasma glucose, 2 h postprandial plasma glucose and glycated hemoglobin. Lipid metabolic markers including serum total cholesterol, triglycerides (TG), high-density lipoprotein (HDL), low-density lipoprotein (LDL), and free fatty acids (FFA) were tested. The diagnosis of hypertension was systolic blood pressure over 140 mmHg or diastolic blood pressure over 90 mmHg (17). Diabetes was defined as fasting plasma glucose levels ≥ 7.0mmol/L or 2 h postprandial plasma glucose ≥11.1 mmol/L (18, 19).

### Measurement and Diagnosis of OSAS

We used overnight polysomnography (SOMNOlab2, Weinmann, Germany) in our study to assess whether the patients had OSAS. The result of the overnight polysomnography was presented as an apnea hypopnea index (AHI), measuring the total number of apneas and hypopneas per hour of sleep. It is considered the gold standard for the diagnosis of OSAS and is widely used in clinical practice (20). Based on the AHI values, OSAS was divided into three categories which were mild OSAS (AHI 5 to 15 events/h), moderate OSAS (AHI 15 to 30 events/h), and severe OSAS (AHI ≥30 events/h) (21). One day before the polysomnography was performed, patients were instructed to stop taking sedative and hypnotic agents.

### Examination of the Abdominal Fat Distribution

We utilized a fat measurement device (DHS-2000, Omron, Japan) to measure the abdominal fat distribution, which uses the theory of bioelectrical impedance analyses (BIA) to measure the area of every abdominal component. The cross-sectional image at the L3 level of the lumbar vertebra was selected to measure the abdominal fat distribution, and the results were exhibited in square centimeters. Moreover, this measurement does not use radiation and can be repeated multiple times.

### Statistical Analyses

Normally distributed continuous data were presented as means ± standard deviations (SD), nonnormally distributed data were presented as medians (quartile, third quartile), and categorical variables were presented as numbers (percentages). Normally distributed continuous data were compared using the independent samples *t*-test. Nonnormally distributed continuous data were compared using the Mann–Whitney *U* test. Pearson’s *χ* ^2^ test or Fisher’s exact test were used to examine the differences between categorical variables. ANOVA approach for statistical comparisons of different degree OSAS. Univariate and multivariate logistic regression analyses were used to evaluate the risk factors for OSAS. *p* < 0.05 was considered statistically significant. All statistical analyses were performed using SPSS version 22 (IBM Corp, Armonk, NY, USA).

## Results

### General Clinical Characteristics of the Participants

**Figure 1:**
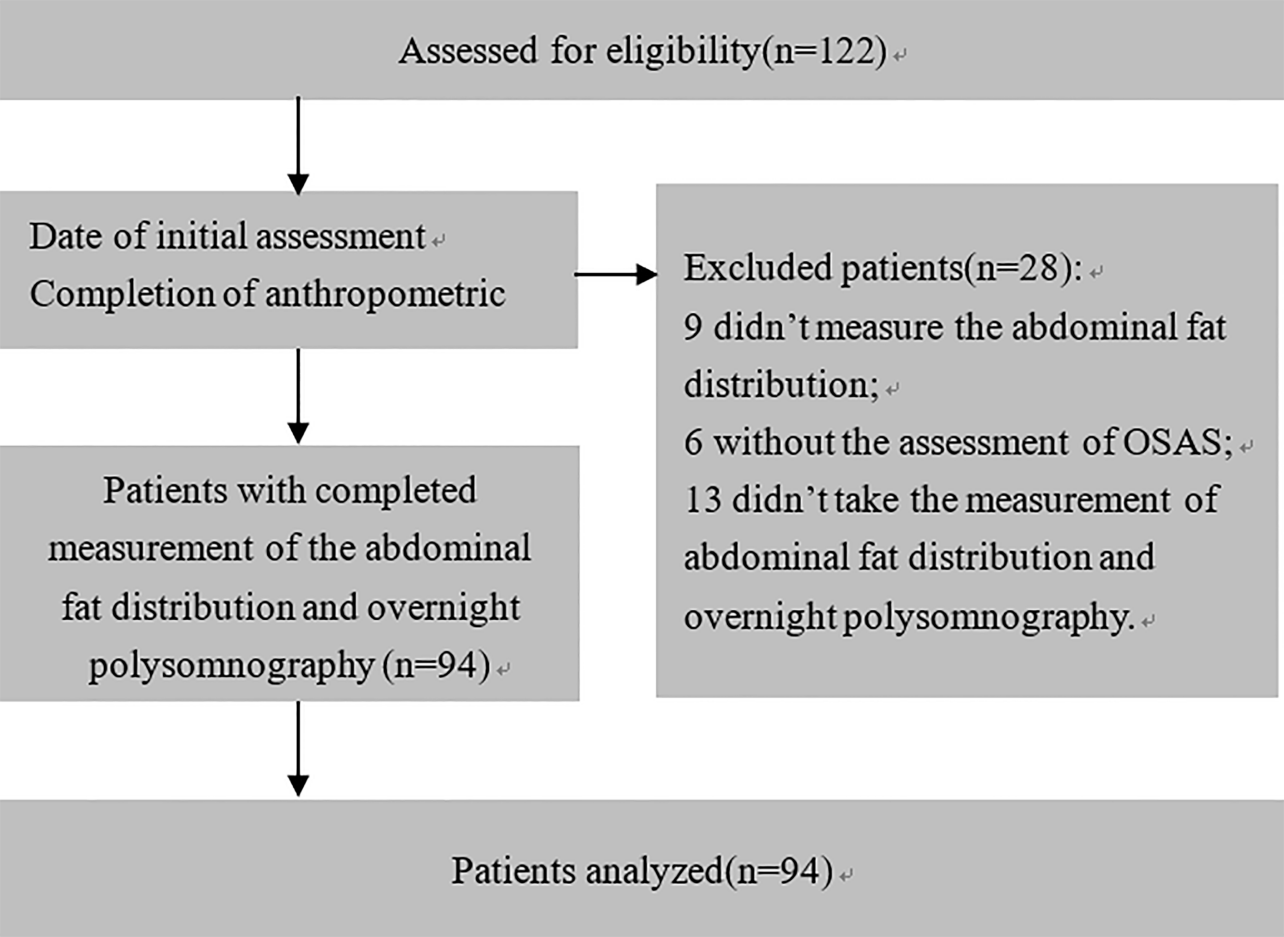
Patient flow diagram showing patient selection. A total of 28 patients were excluded for technical reasons: 9 patients did not have an examination of the abdominal fat distribution, 6 patients without the assessment of OSAS, and 13 patients without neither the examination of abdominal fat distribution nor the assessment of OSAS.

**Figure 2:**
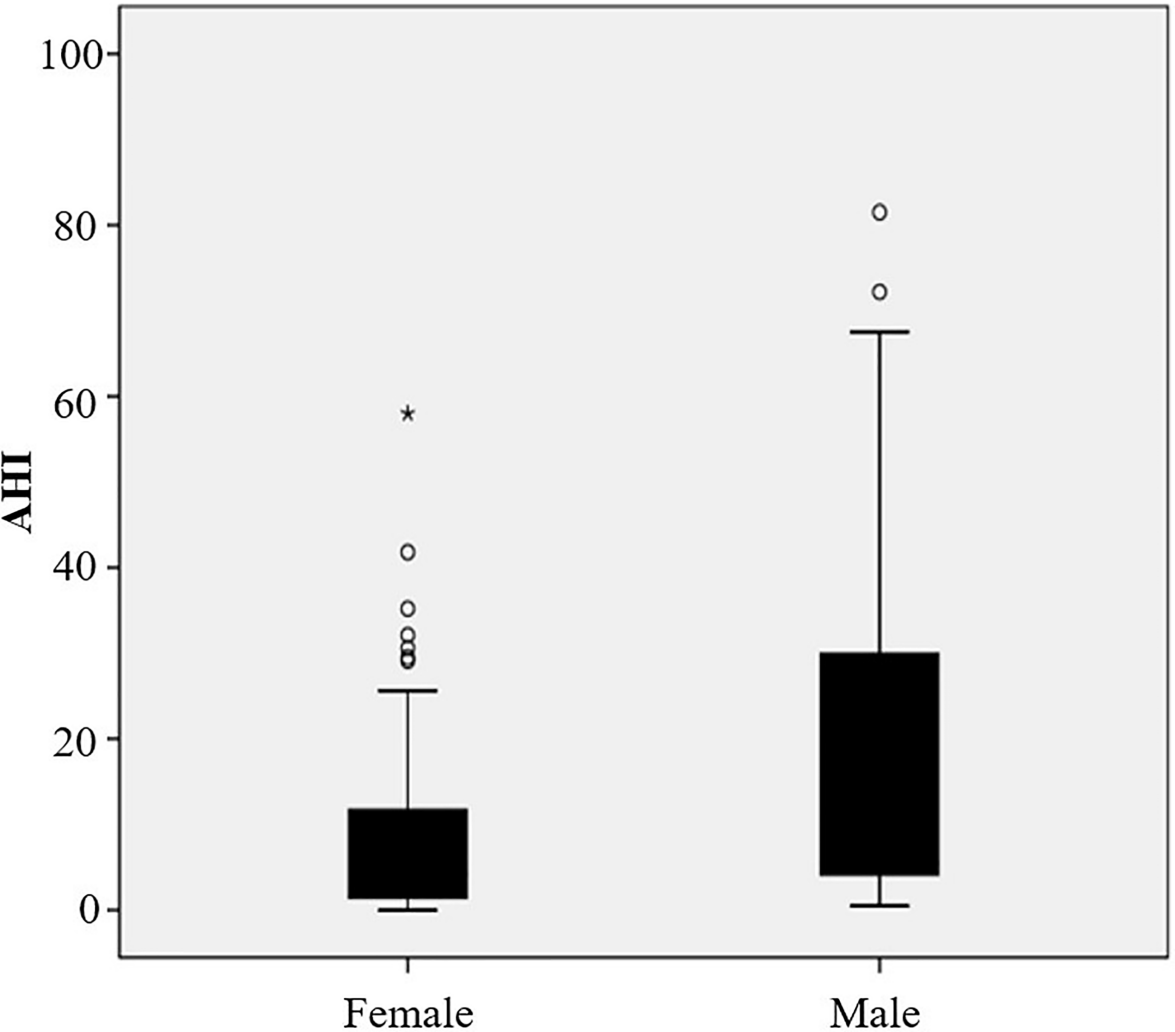
The value of AHI in female and male patients (*p* = 0.014).

**Figure 3:**
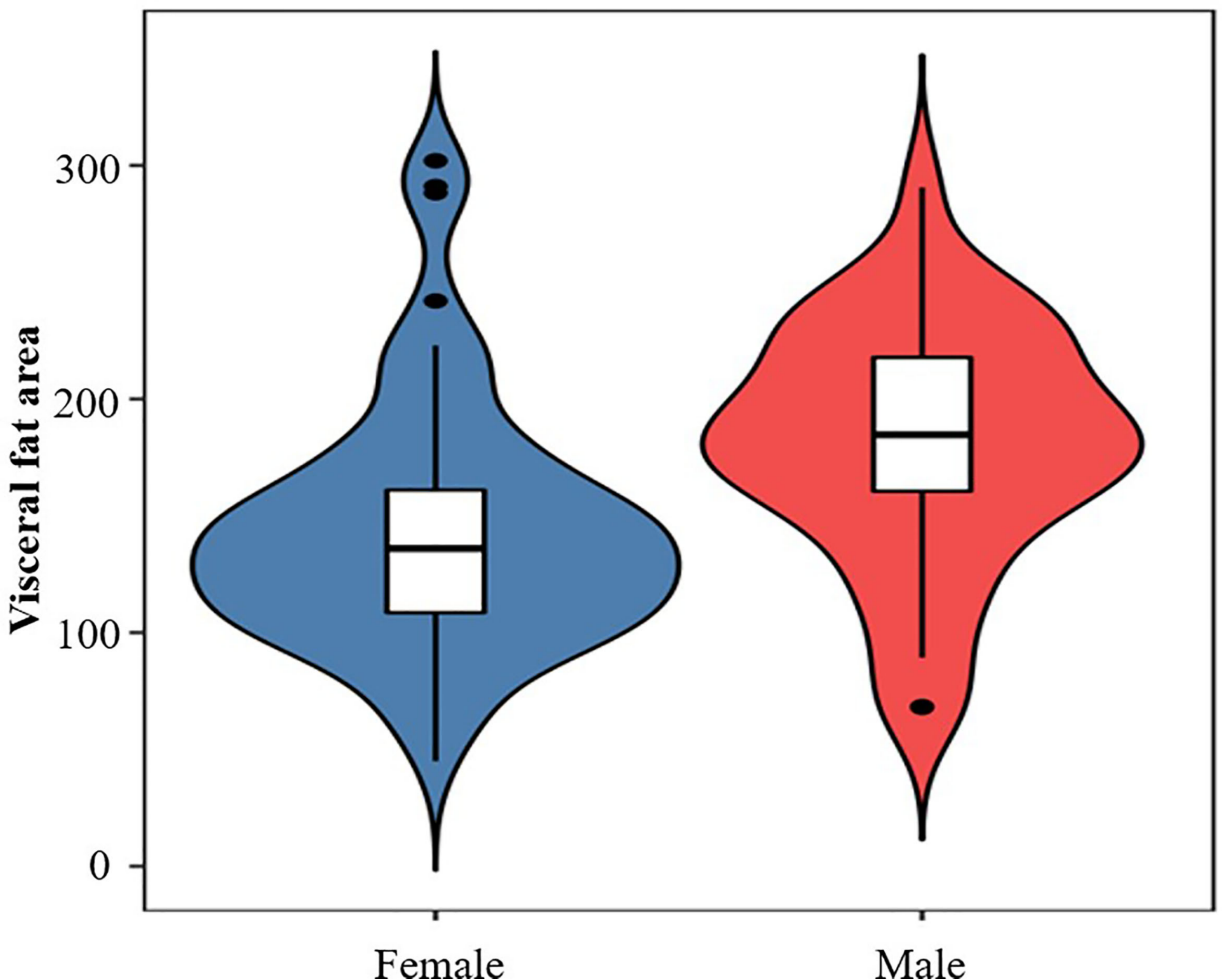
The visceral fat area in female and male patients (*p* = 0.002).

Of 122 consecutive patients, 28 were excluded because they lacked the result of abdominal fat distribution or overnight polysomnography, as shown in **Figure 1**. Ninety-four obese patients were enrolled in this study with a mean (SD) age of 32.44 (11.81) years and mean (SD) BMI of 38.88 (5.87) kg/m^2^. The detailed baseline characteristics are shown in **Table 1**. The overall incidence of OSAS was 51.06%. The majority of patients were women (62.77%), but the incidence of OSAS in women was slightly lower than in men (44.07% vs. 62.86%, *p* = 0.078). Furthermore, the AHI in men was significantly higher than in women (5.50; IQR, 3.80–30.6 vs. 4.2; IQR, 1.4–12 events/h, *p* = 0.014); the results are shown in **Figure 2**. Meanwhile, men had a significantly higher visceral fat area compared with women (180.29 ± 51.64 vs. 143.88 ± 53.42 cm^2^, *p* = 0.002) as illustrated in **Figure 3**. In addition, patients with hypertension (*p* = 0.030) or diabetes (*p* = 0.019) also exhibited a significantly higher AHI when compared with the patients without corresponding complications.

**Table 1:** Patient demographic characteristics and correlation with AHI.

	Total (*N* = 94)	*R*	*p*
Age (year)	31.00 (24.00–37.00)	0.268	0.001^*^
Sex			0.014^*^
Male	35 (37.23%)		
Female	59 (62.77%)		
Height (m)	1.68 (0.09)	0.155	0.137
Weight (kg)	103.40 (92.60–124.25)	0.197	0.056
BMI (kg/m^2^)	38.00 (34.59-41.92)	0.306	0.003^*^
Neck circumference (cm)	42.35 (4.71)	0.380	<0.001^*^
Waist circumference (cm)	117.44 (13.41)	0.328	0.002^*^
Hip circumference (cm)	120.26 (11.41)	0.189	0.086
OSAS			<0.001^*^
None	46 (48.94%)		
Mild	24 (25.53%)		
Moderate	10 (10.64%)		
Severe	14 (14.89%)		
Visceral fat area (cm^2^)	157.44 (55.39)	0.420	<0.001^*^
Subcutaneous fat area (cm^2^)	411.99 (115.46)	0.010	0.094
Diabetes			0.019^*^
Yes	30 (31.91%)		
No	64 (68.09%)		
Hypertension			0.030^*^
Yes	69 (73.40%)		
No	25 (26.60%)		
Triglyceride (mmol/L)	1.64 (1.19–2.42)	0.135	0.200
Cholesterol (mmol/L)	4.66 (0.91)	0.081	0.443
LDL (mmol/L)	2.84 (0.77)	0.142	0.179
HDL (mmol/L)	1.01 (0.24)	0.170	0.106
FFA (mmol/L)	0.66 (0.23)	0.052	0.625

OSAS, obstructive sleep apnea syndrome; AHI, apnea hypopnea index; BMI, body mass index; HDL, high-density lipoprotein; LDL, low-density lipoprotein; FFA, free fatty acids.

^*^p < 0.05, statistically significant.

### Comparison of Different Degree OSAS

**Figure 4:**
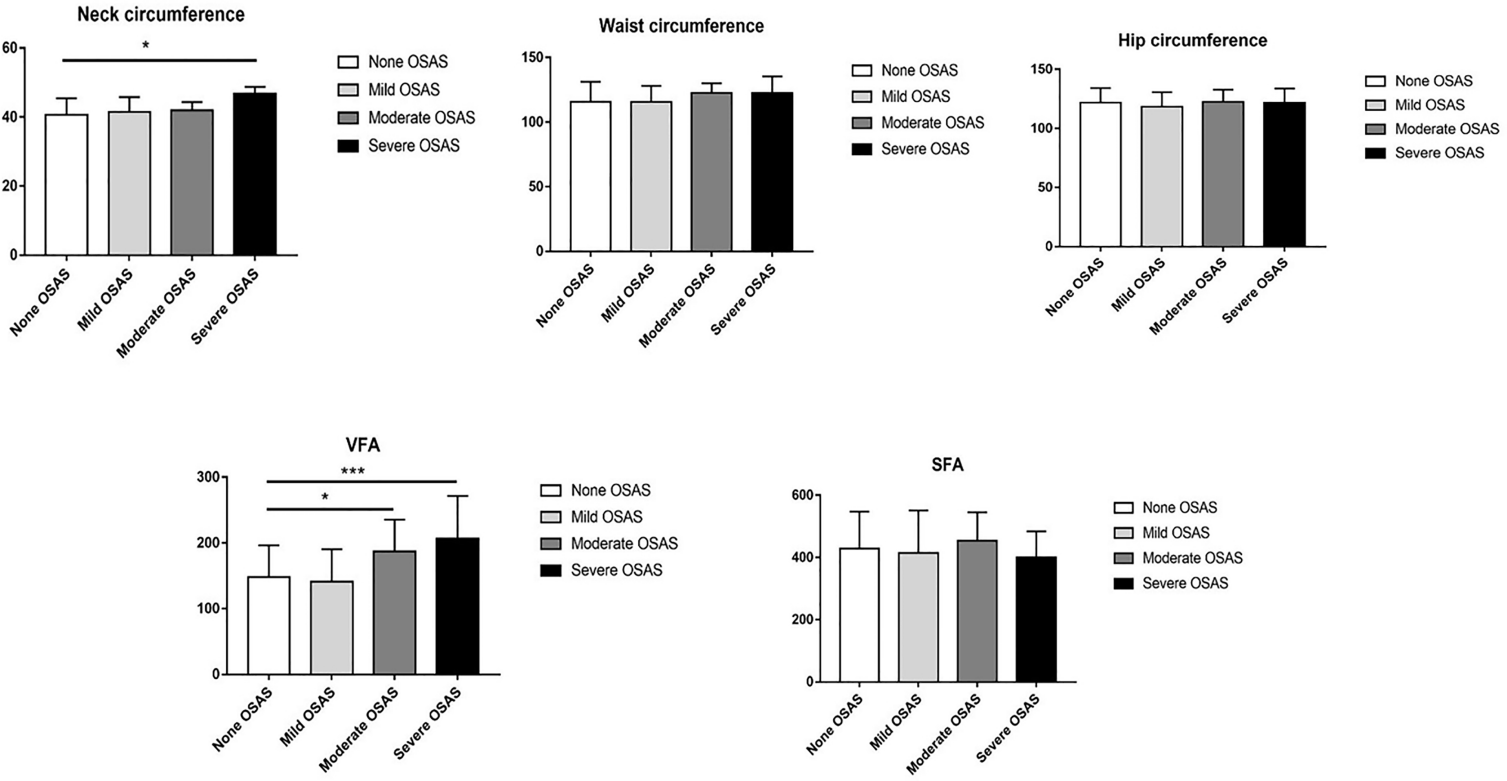
Comparison of neck circumference, waist circumference, hip circumference, visceral fat area (VFA), and subcutaneous fat area (SFA) between different severity OSAS. *p < 0.05; ***p < 0.001.

Among those patients with OSAS, 24 (25.53%) patients had mild OSAS, 10 (10.64%) had moderate OSAS, and 14 (14.89%) had severe OSAS. According to the results of ANOVA test, we found the neck circumference of severe OSAS group was significantly higher than non-OSAS group (*p* < 0.05), as shown in **Figure 4**. Though the results were not significant, the mean neck circumference of mild OSAS and moderate OSAS group were all higher than the non-OSAS group (all *p* > 0.05). Compared with the non-OSAS group, the visceral fat area of the moderate (*p* < 0.01) and severe OSAS group (*p* < 0.001) were significantly higher as shown in **Figure 3**.

### Association of Abdominal Fat Distribution and AHI

Correlation analysis indicated that AHI was significantly positively associated with BMI, neck circumference, waist circumference, and visceral fat area (*r* = 0.306, *p* = 0.003; *r* = 0.380, *p* < 0.001; *r* = 0.328, *p* = 0.002; *r* = 0.420, *p* < 0.001) but was not associated with subcutaneous fat area (*p* = 0.094). Additionally, marker of lipid metabolism including triglyceride, cholesterol, LDL, HDL, and FFA had no significant association with AHI (**Table 1**).

### Univariate Analysis of Factors Associated With OSAS

**Figure 5:**
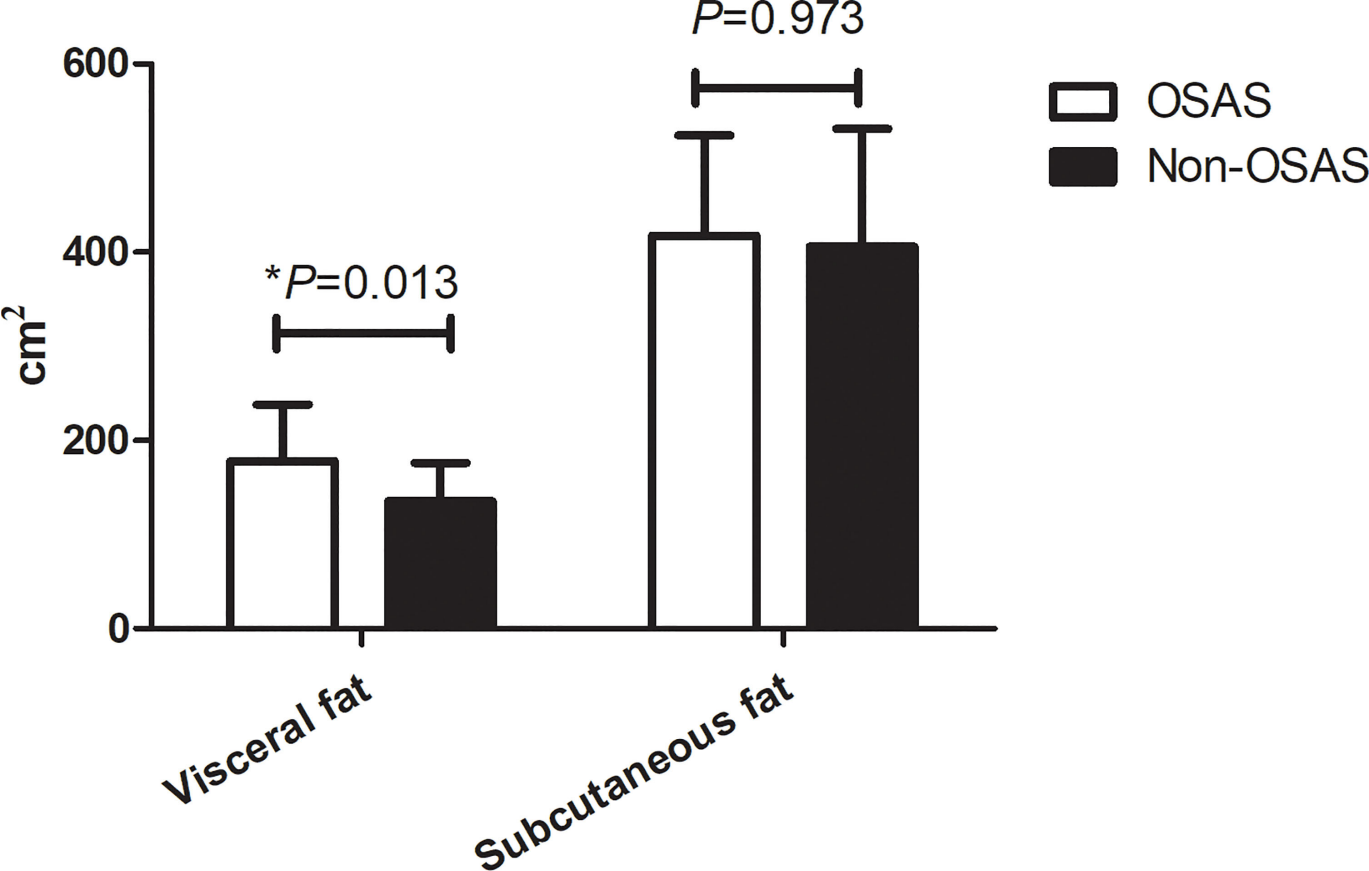
Comparison of abdominal fat distribution in obese patients with or without OSAS. *p < 0.05.

Based on the AHI values, the cohort was divided into two groups: the non-OSAS (AHI <5 events/h) and the OSAS group (AHI ≥5 events/h). Compared with the non-OSAS group, patients with OSAS had a significantly higher age (33.00; IQR, 27.25–37.00 vs. 28.00; IQR, 23.00–34.75 years, *p* = 0.007). The fasting plasma glucose, 2 h postprandial plasma glucose, and glycated hemoglobin were all significantly higher in the OSAS group than in the non-OSAS group (all *p* < 0.05). However, the HDL of the OSAS group was significantly lower than that of the non-OSAS group (0.96 ± 0.21 vs. 1.06 ± 0.27 mmol/L, *p* = 0.008) while the waist circumference of the OSAS group was significantly higher (121.21 ± 12.69 vs. 113.08 ± 13.03 cm, *p* = 0.043). Although the results did not reach statistical significance, the mean neck circumference and hip circumference of the OSAS group were higher compared with those of the non-OSAS group, as are shown in **Table 2**. Interestingly, in the OSAS group, the visceral fat area was significantly higher than in the non-OSAS group (178.28 ± 59.89 vs. 135.68 ± 40.58 cm^2^, *p* = 0.013) while no significant differences were found in the subcutaneous fat area and BMI between groups (417.56 ± 106.48 vs. 406.18 ± 125.07 cm^2^, *p* = 0.970), as presented in **Figure 5**. Overall, obese patients with OSAS had higher visceral adiposity rather than subcutaneous fat. Glucose-lipid metabolism disorders were more severe in obese patients with OSAS.

**Table 2:** Univariate analysis of factors associated with OSAS.

	OSAS (*N* = 48)	Non-OSAS (*N* = 46)	*p*-value
Age (year)	33.00 (27.25–37.00)	28 (23.00–34.75)	0.007^*^
Sex			0.078
Male	22 (62.86%)	13 (37.14%)	
Female	26 (44.07%)	33 (55.93%)	
Height (m)	1.70 (0.08)	1.66 (0.10)	0.092
Weight (kg)	115.20 (98.15–131.08)	99.95 (89.70–118.00)	0.078
BMI (kg/m^2^)	39.55 (35.40–45.25)	37.18 (33.98–40.37)	0.257
Neck circumference (cm)	43.72 (4.66)	40.77 (4.29)	0.132
Waist circumference (cm)	121.21 (12.69)	113.08 (13.03)	0.043^*^
Hip circumference (cm)	121.40 (12.39)	118.93 (10.18)	0.559
Systolic pressure (mmHg)	143.35 (19.43)	135.98 (21.79)	0.146
Diastolic pressure (mmHg)	86.19 (12.85)	81.17 (11.82)	0.116
Visceral fat area (cm^2^)	178.28 (59.89)	135.68 (40.58)	0.013^*^
Subcutaneous fat area (cm^2^)	417.56 (106.48)	406.18 (125.07)	0.970
Fast plasma glucose (mmol/L)	6.05 (5.13–8.18)	5.2 (4.88–6.00)	0.007^*^
2 h postprandial plasma glucose (mmol/L)	9.50 (7.18–16.08)	7.75 (6.50–9.50)	0.012^*^
Glycated hemoglobin (%)	6.89 (5.70–8.00)	5.90 (5.60–6.23)	0.001^*^
Triglyceride (mmol/L)	1.78 (1.31–2.71)	1.45 (1.14–2.38)	0.122
Cholesterol (mmol/L)	4.63 (0.93)	4.69 (0.90)	0.052
LDL	2.79 (0.76)	2.90 (0.79)	0.698
HDL	0.96 (0.21)	1.06 (0.27)	0.008^*^
FFA	0.67 (0.22)	0.65 (0.24)	0.855

OSAS, obstructive sleep apnea syndrome; AHI, apnea hypopnea index; BMI, body mass index; HDL, high-density lipoprotein; LDL, low-density lipoprotein; FFA, free fatty acids.

^*^p < 0.05, statistically significant.

### Results of the Multivariate Analysis

According to the results of the univariate analysis, the factors with *p* < 0.05, including age, waist circumference, visceral fat area, HDL, fast plasma glucose, 2 h postprandial plasma glucose, and glycated hemoglobin, were further analyzed in the multivariate analysis. The results are shown in **Table 3**. Visceral fat area (odds ratio, 1.016; 95% confidence interval, 1.005–1,026; *p* = 0.005) and fasting plasma glucose (odds ratio, 1.618; 95% confidence interval, 1.149–2.278; *p* = 0.006) were found to be the independent risk factors for OSAS.

**Table 3:** Multivariate logistic regression analysis of factors associated with OSAS.

	Odds ratio	95% CI	*p*-value
Visceral fat area	1.016	1.005–1.026	0.005^*^
Fast plasma glucose	1.618	1.149–2.278	0.006^*^

^*^p < 0.05, statistically significant.

## Discussion

The findings of our study revealed that the prevalence of OSAS was higher in obese patients and was associated with the deposition of abdominal visceral adipose tissue. Furthermore, abdominal visceral adipose accumulation was an independent risk factor for OSAS.

The incidence of obesity is increasing year by year and has become a public health issue. Obesity is strongly linked with many metabolic diseases such as type 2 diabetes, hypertension, cardiovascular diseases, dyslipidemia, nonalcoholic fatty liver disease, chronic kidney disease, obstructive sleep apnea, and hypoventilation syndrome (22, 23). Obesity is closely related to OSAS. Insulin resistance caused by obesity as well as low levels of vitamin D (Vit D) presented in obesity are also a risk factor of OSAS (13, 24, 25). Based on the association between levels of Vit D and OSAS, there was a study that investigated whether Vit D supplementation can improve the prognosis of mild OSAS and found that Vit D supplementation had a positive effect which could significantly decrease the AHI value of the patients (26).

According to the results of our study, over half of the obese patients had OSAS and male obese patients demonstrated a higher incidence of OSAS. Therefore, the management of weight may play a crucial role and weight loss has been shown to be an effective treatment for OSAS (27). In a study by Del Genio et al., OSAS patients were followed up for 5 years, and it was reported that sleeve gastrectomy could improve OSAS (28). However, as is known, fat distribution is more important than the total amount of body fat in predicting obesity-causing complications (29). The crosstalk between fat distribution and OSAS has not been fully clarified. No study has explored the relationship between fat distribution and OSAS. We underwent this study with the question: whether abdominal fat distribution affects the occurrence of OSAS.

BMI is the basis for the World Health Organization classification of obesity and has been used to assess the degree of obesity. However, due to individual differences, people may have the same BMI but a different distribution of fat and muscle tissue. Hence, BMI alone cannot accurately reflect the distribution of body fat in obese patients in the clinic (30). In this study, we analyzed the association between BMI and OSAS. The results were not statistically significant, possibly because the enrolled patients were Asians, who more commonly exhibit central obesity with a normal BMI (31). Central obesity may also be one of the reasons why waist circumference was significantly associated with OSAS, but hip and neck circumference were not. In contrast to some studies that reported that neck circumference was associated with the incidence of OSAS [20], univariate analysis in our study did not show a significant relationship between neck circumference and OSAS. The main reason for this may be that we defined AHI <5 events/h as the group standard whereas many other studies designated AHI <15 events/h as the cutoff. Additionally, when we further compared the different degrees of OSAS, we found that the neck circumference of severe OSAS patients was significantly higher than that of non-OSAS patients.

Furthermore, on measuring the abdominal adipose tissue distribution, we found that visceral adipose tissue was significantly associated with OSAS. Abdominal adiposity is known to be associated with decreased lung volumes and hypoventilation (32). Increased abdominal visceral adiposity decreases the lung volumes, including the functional residual volume, which reduces traction on the pharynx, and this may subsequently result in increased pharyngeal collapsibility and, thus, OSAS (15). In addition, abdominal fat distribution was assessed by bioelectrical impedance analyses (BIA). This was the first study to evaluate the relationship between OSAS and abdominal fat distribution. The results of our study were similar to those of a study by Turnbull, who explored the relationship between fat distribution assessed by magnetic resonance imaging (MRI) and OSAS (14). In his study, abdominal visceral fat at the L2–3 level was significantly associated with OSAS (*p* = 0.02). Similarly, Kritikou et al. used computer tomography (CT) to assess visceral fat and proved that visceral adiposity was significantly associated with OSAS (33). Hence, in the assessment of fat distribution, BIA can have the same effect as MRI and CT. Compared with CT and MR, BIA is relatively cheap, portable, has no radiation, and consequently, may be more suitable for the screening of OSAS. Overall, the findings of our study revealed that the prevalence of OSAS was higher in obese patients and was associated with the deposition of abdominal visceral adipose tissue which was measured by a kind of simple and accurate measurement method. Also, abdominal visceral adipose accumulation was an independent risk factor for OSAS.

Several studies have shown that OSAS is significantly associated with T2DM, and a linear association has been found (34). In our study, we also discovered that the levels of fasting plasma glucose and glycated hemoglobin were significantly higher in obese patients with OSAS than in patients without OSAS. Besides blood glucose, OSAS is also associated with other components of metabolic syndrome, such as blood lipids and blood pressure (35). A previous study has revealed that serum triglyceride levels were significantly associated with OSAS (36). However, our study did not present a similar result, which may be due to a difference in the cohort.

However, our study has also some limitations. Firstly, the sample size is comparatively small because the measurement of OSAS requires special equipment and conditions, and not everyone consented to the examination. Secondly, our study is a retrospective study; we did not investigate whether an improvement in obesity and the reduction of abdominal fat can lead to an improvement in OSAS. There is a lack of data. Larger-scale and well-designed randomized controlled trials are necessary in the future.

## Conclusion

The prevalence of OSAS was high in obese patients and was associated with the deposition of abdominal fat, especially visceral adipose tissue. Abdominal visceral adipose accumulation but not subcutaneous fat deposition was an independent risk factor for OSAS in obese patients, which may have important clinical significance in the assessment and treatment of OSAS. However, a larger-scale study would be needed in the future.

## Data Availability Statement

The raw data supporting the conclusions of this article will be made available by the authors, without undue reservation.

## Ethics Statement

The studies involving human participants were reviewed and approved by the Shanghai Tenth People’s Hospital. The patients/participants provided their written informed consent to participate in this study.

## Author Contributions

BM and YL performed the experiment. XW and TU helped in drafting the manuscript. LD and SW participated in the data collection. HM and DZ helped in the statistical analysis. LL and SQ designed the study. All authors listed have made a substantial, direct, and intellectual contribution to the work and approved it for publication.

## Funding

The research was supported by the Climbing Talent Program of the Shanghai Tenth People’s Hospital (2021SYPDRC047).

## Conflict of Interest

The authors declare that the research was conducted in the absence of any commercial or financial relationships that could be construed as a potential conflict of interest.

## Publisher’s Note

All claims expressed in this article are solely those of the authors and do not necessarily represent those of their affiliated organizations, or those of the publisher, the editors and the reviewers. Any product that may be evaluated in this article, or claim that may be made by its manufacturer, is not guaranteed or endorsed by the publisher.
